# Pre-analytical errors in a high-volume Bangladeshi diagnostic centre: Prevalence, workload impact, and mitigation strategies

**DOI:** 10.1371/journal.pone.0341908

**Published:** 2026-03-04

**Authors:** Indrajit Sarkar, Kona Rani Sarkar

**Affiliations:** 1 Department of Biochemistry and Immunology, Popular Diagnostic Centre Ltd., Dhaka, Bangladesh; 2 Department of Public Health, Atish Dipankar University of Science & Technology, Dhaka, Bangladesh; 3 Department of Economics, Begum Rokeya University, Rangpur, Bangladesh; University of Waterloo, CANADA

## Abstract

**Background:**

Pre-analytical errors are the most frequent cause of laboratory mistakes, accounting for nearly half of all diagnostic inaccuracies worldwide. These errors can invalidate test results, delay clinical decisions, and waste valuable healthcare resources, particularly in resource-limited, high-volume diagnostic laboratories. This study aimed to assess the prevalence, contributing factors, and severity of pre-analytical errors in a large diagnostic centre in Bangladesh.

**Methods:**

An observational, cross-sectional study was conducted over two months in the Biochemistry and Immunology Laboratories of a high-volume diagnostic centre in Dhaka, Bangladesh. Data from 195 documented pre-analytical errors and a structured survey of 27 laboratory staff were analysed. Errors were classified into minor, moderate, or major using definitions adapted from ISO 15189:2022 and WHO guidelines. Descriptive statistics and Chi-square tests were performed to explore associations between workload level (≥ 931 samples/day) and error frequency, with p < 0.05 considered statistically significant.

**Results:**

The most frequent errors were sample misplacement (38.5%) and incorrect labelling (17.9%). The sample collection (42.6%) and pick-and-drop (38.5%) units contributed the majority of errors. Morning shifts (65.1%) and high-workload days (70.8%) showed higher error frequencies, with a statistically significant association between workload and error occurrence (χ² = 121.093, p < 0.001). Major errors accounted for 37.4% of incidents.

**Conclusion:**

Pre-analytical errors remain a critical threat to diagnostic accuracy in resource-limited laboratories. Improving workflow organization, implementing barcoding and automation, and strengthening staff training and workload management can substantially reduce error rates and enhance patient safety in high-throughput clinical settings.

## Introduction

Clinical laboratories are essential facilities within the healthcare continuum. However, the pre-analytical phase is, by far, the most error-prone component of laboratory medicine, with 46–68% of laboratory errors occurring during this phase [1–4], a finding consistent with broader reviews of medical errors in laboratory diagnostics [5]. The occurrence of problems such as sample misplacement, incorrect labeling, and incorrect test order can jeopardize the accuracy of clinical diagnosis, leading to unacceptable delays in and increased costs of patient treatment [6–8].

In low-resourced settings, these challenges are compounded by factors such as high workloads, inadequate staffing, and inefficient workflow processes [9–11]. Laboratories in such environments often lack automation and must depend on manual procedures, which increase the risk of variability and human error. Furthermore, the absence of continuous training and the use of outdated Standard Operating Procedures (SOPs) often exacerbate these challenges [9].

International standards such as “*ISO 15189:2022*” remind laboratories of the importance of continuously monitoring and striving to improve all pre-analytical processes in order to minimize the risks described [12,13]. Compliance with such standards facilitates traceability, accountability, and continuous improvement, making quality indicators a vital part of laboratory performance monitoring.

With this in mind, the present study sought to determine types, frequency, and contributing factors of pre-analytical errors in a high-volume diagnostic center’s biochemistry and immunology laboratories in Bangladesh. The focus was to characterize error incidents and relate them to workload and shifts. By identifying patterns and operational determinants, this research also seeks to support evidence-based policy recommendations for laboratory quality management in low-resource contexts.

## Methods

### Operational definitions of error types

Pre-analytical errors were categorized and defined as follows:

- Sample Misplacement: A sample that was physically received in the laboratory but not properly accepted in the Laboratory Information System (LIS), leading to tracking failures. This occurs when: (1) Sample pick-and-drop staff transport samples without completing LIS acceptance; (2) Automated analyzers cannot process unaccepted samples, leaving them in unknown locations; (3) Samples are stored in incorrect racks without proper processing.
- Sample Tube Missing: A sample that was recorded as collected but never physically received in the laboratory.
- Incorrect Labeling: A sample with missing, incomplete, or mismatched patient identification information.
- Incorrect Test Order: Errors occurring during manual test entry into analyzers, typically during LIS incompatibility or system outages. This includes situations where laboratory staff manually enter test requests and mistakenly select or run incorrect tests due to the absence of automated validation.
- Incorrect Tube: Use of an inappropriate collection container for the requested test.
- Insufficient Sample Volume: Sample quantity inadequate for performing the requested tests.
- Hemolysis and Clotting: Sample quality issues make it unsuitable for analysis. (Hemolysis and clotting were recorded as a combined category because the laboratory’s error log captures these events under a single entry.)
- Barcode Error: Failure in barcode scanning or printing affecting sample identification.
- Duplicate Test Order: Same test requested multiple times without clinical justification.
- Host Communication Error: Failure in electronic data transfer between systems.

Errors were classified into three severity levels based on their potential clinical impact, with definitions adapted from *ISO 15189:2022* and WHO guidelines [12–14], following established principles for using quality indicators to monitor laboratory errors [15]:

- Minor: Errors causing a minor delay but not resulting in sample rejection.
- Moderate: Errors requiring corrective action and causing a moderate reporting delay, with a low risk of significant misdiagnosis.
- Major: Errors leading to sample rejection, a lengthy reporting delay, or a potential diagnostic error.

### Ethics statement

Ethical approval for this study was obtained from the Ethical Review Committee of Atish Dipankar University of Science and Technology (ADUST). Popular Diagnostic Centre Ltd. provided institutional permission to access anonymized pre-analytical error data and to administer the staff survey. Written informed consent was obtained from all survey respondents. For the retrospective error data analysis, all data were fully anonymized before access, and the requirement for patient consent was waived because no identifiable personal information was used.

### Study design

This descriptive cross-sectional study was conducted over a two-month period at the Biochemistry and Immunology Laboratories of a high-volume diagnostic centre in Dhaka, Bangladesh. Data for this study were derived from all documented pre-analytical errors that met the predefined classification criteria, and survey responses from the laboratory staff involved in the pre-analytical phase. Data for this study were collected and accessed for research purposes from November 1, 2024, to December 31, 2024.

The diagnostic centre processes more than 2000 samples daily across multiple departments, including biochemistry, immunology, microbiology, clinical pathology, histopathology, and PCR. For this study, only the biochemistry and immunology sections, which together handle approximately 800–1200 samples per day, were included in the analysis. The pre-analytical phase at the centre includes sample reception, barcode generation (manual printing and labeling), phlebotomy, and transportation to analytical sections. Each stage follows internal standard operating procedures (SOPs) aligned with ISO 15189 standards, although deviations were occasionally observed.

### Subject and data collection

All errors that occurred during the study period and met the pre-defined error classification criteria were included in the analysis. Additionally, a structured questionnaire was administered to 27 staff members directly involved in pre-analytical processes across four units: (1) Biochemistry and Immunology (sample reception, sorting, and preparation), (2) Sample Collection (phlebotomy and initial specimen handling), (3) Entry Counter (patient registration and test order entry), and (4) Sample Pick-and-Drop (specimen transportation between units). All surveyed staff participated exclusively in pre-analytical activities. The questionnaire focused on perceived workload, staff experience, training adequacy, and common operational bottlenecks.

To ensure reliability, the data collection process was supervised by a designated quality control officer. Data sources were cross-checked weekly to confirm consistency between paper records and Laboratory Information System (LIS) entries. The use of triangulated data sources helped minimize recall bias and enhanced the internal validity of findings.

Data were collected from three primary sources: (1) daily pre-analytical error logs, which documented the error type, severity, and department of origin; (2) the Laboratory Information System (LIS), which provided total daily sample volume; and (3) structured staff surveys assessing perceptions of workload and process issues.

Each reported incident was verified by a quality officer to ensure that the classification was accurate and non-duplicated. In case of uncertainty, a consensus meeting between supervisors and laboratory technologists was used to validate the record.

The independent variables were daily workload, categorized as high (≥931 samples) or low, based on operational thresholds established by the laboratory**,** and shift timing (morning or evening). The dependent variables were the type and severity of pre-analytical errors.

To ensure consistency, data entry and error coding were double-checked by two independent reviewers. Cross-verification between reviewers ensured consistency in classification, and discrepancies were resolved by discussion. This approach minimized subjective interpretation and enhanced reproducibility.

### Statistical analysis

Data were analyzed using IBM SPSS Statistics for Windows, Version 26.0 (IBM Corp., Armonk, NY, USA). Descriptive statistics (frequencies and percentages) were used to summarize the distributions of error types, locations, severity, and shift timing. The chi-square test was used to assess the association between daily workload (high vs. low) and error occurrence. Statistical significance was established at a threshold of *p* < 0.05.

To enhance analytical rigor, error rates were also compared by unit and shift duration. Cross-tabulations were created to identify overlapping factors contributing to error occurrence. Graphical visualizations were prepared to illustrate relationships between workload intensity and error frequency trends.

## Results

### Frequency and distribution of errors

Through systematic daily monitoring of both electronic and manual laboratory error logs, and cross-referencing with the Laboratory Information System (LIS), a total of 195 pre-analytical errors were recorded. The most common error was sample misplacement (38.5%), followed by incorrect labeling (17.9%) and incorrect test orders (13.8%). (Table 1). Other, less frequent but operationally significant errors included incorrect tubes and insufficient sample volume, which collectively represented around 15% of all incidents.

**Table 1:** Distribution of pre-analytical error types (n = 195).

Type of pre-analytical error		
Pre-analytical error	Frequency	Percent
Sample Misplacement	75	38.5
Incorrect Labeling	35	17.9
Incorrect Test Order	27	13.8
Incorrect Tube	15	7.7
Insufficient Sample Volume	14	7.2
Hemolysis and Clotting	10	5.1
Sample Tube Missing	7	3.6
Barcode Error	5	2.6
Duplicate Test Order	4	2.1
Host Communication Error	3	1.5
Total	195	100.0

**Fig 1:**
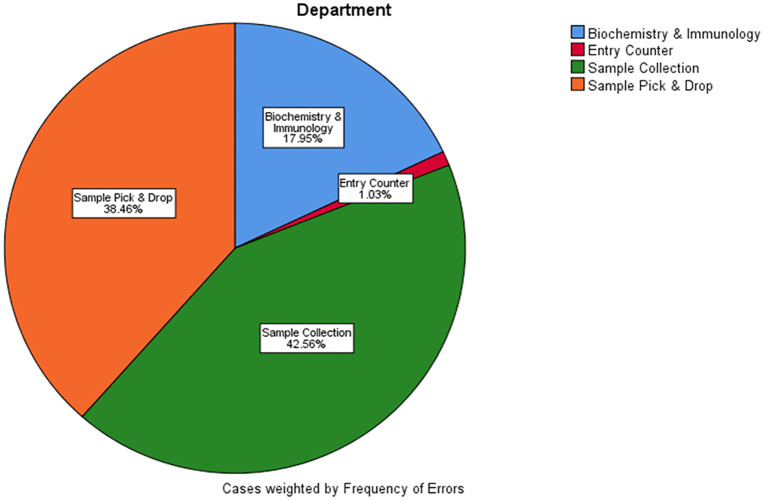
Distribution of recorded pre-analytical errors by unit.

The sample collection unit had the highest proportion of errors (42.6%), followed closely by the sample pick-and-drop unit (38.5%) (Fig 1). Errors were more frequent during morning shifts (65.1%) compared with evening shifts (34.9%)(Table 2).

**Table 2:** Distribution of pre-analytical errors by shift.

Shift timing	Total errors (n)	Percentage (%)
Morning	127	65.1
Evening	68	34.9
Total	195	100.0

Survey crosstab analysis showed that 94.1% (16/17) of morning-shift staff reported a heavier workload during their shift, compared with 60.0% (6/10) of evening-shift staff (S1 Table), supporting the higher error frequency observed in morning hours. Errors occurred across multiple units, indicating that pre-analytical issues were not confined to a single step of the workflow.

Severity of Errors: In terms of clinical impact, major errors accounted for 37.4% of recorded incidents, moderate errors accounted for 34.4%, and minor errors made up 28.2%. The sample collection unit had the highest recorded incidence of major errors (48.2%), while the biochemistry and immunology department had the highest proportion of recorded moderate errors (77.1%). Table 3 presents the breakdown of error severity by unit, including the absolute counts underlying the reported percentages.

**Table 3:** Severity of errors by unit.

Unit	Minor (%)	Moderate (%)	Major (%)	Total errors (n)
Sample Collection	12%	39.8%	48.2%	83
Sample Pick and Drop	49.3%	6.7%	44%	75
Biochemistry and Immunology	22.9%	77.1%	0%	35
Entry Counter	0%	100%	0%	2
Total	28.2%	34.4%	37.4%	195

**Fig 2:**
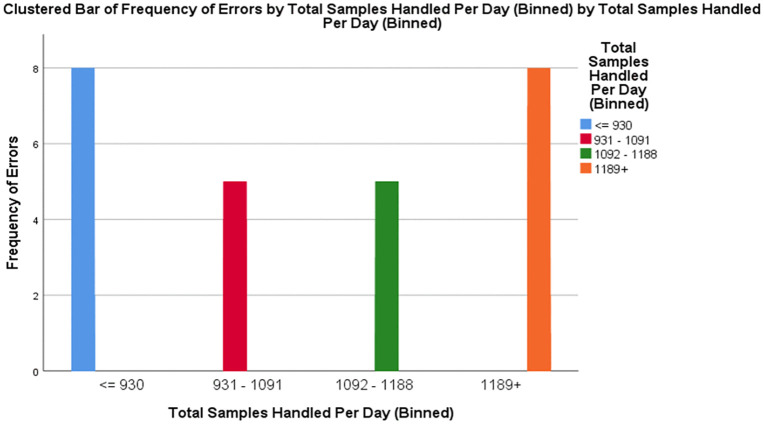
Association between daily workload and pre-analytical error frequency.

Workload and Error Occurrence: Out of all errors, 70.8% occurred on days of high workload (≥931 samples), with 29.2% occurring on days with low workload. The chi-square test showed a statistically significant correlation between workload and the frequency of errors (χ² = 121.093, p < 0.001) (Fig 2).

## Discussion

The results of this research indicate that sample misplacement and incorrect labeling are the major sources of pre-analytical errors in a high-volume diagnostic setting in Bangladesh, which is in agreement with other low- and middle-income countries [9,16,17].

These findings emphasize that the pre-analytical stage represents a critical point in the testing process within this setting. Previous studies have shown that human interaction, manual documentation, and limited automation increase pre-analytical variability [3]; the patterns observed in our setting appear consistent with these findings.

In the studied facility, barcoding and sample tracking processes are only partially integrated, requiring manual printing and attachment of barcodes to tubes. Such manual steps may increase the likelihood of identification discrepancies.

Our observations suggest that operational priorities may emphasize rapid sample processing, which can place pressure on pre-analytical workflows and potentially contribute to errors when quality safeguards are inconsistently applied. This underscores the need for a balanced approach where efficiency targets are matched with quality safeguards through standardized monitoring and staff accountability.

The clustering of errors in the sample collection unit emphasizes this phase as a prime candidate for quality improvement initiatives (Fig 1).

Morning shifts generated higher frequencies of errors (65.1%), which is strongly supported by our survey data showing 94.1% of morning-shift staff reported heavier workload pressures. This aligns with studies identifying time pressure and staff fatigue as key contributors to pre-analytical errors [18,19], and with voluntary incident report analyses linking such workflow pressures to diagnostic error [19]. The presence of errors across both the sample collection and pick-and-drop units suggests that several incidents may arise from shared process steps rather than isolated failures in a single unit, reflecting how workload surges and task distribution challenges can influence pre-analytical performance.

These findings collectively indicate that workload pressure is a primary operational driver of pre-analytical errors, consistent with patterns reported globally [8,9].

Previous research has also highlighted that inconsistent supervision during shift handovers allows cumulative errors to go unnoticed until the analytical phase, where correction becomes difficult or impossible [18,19]. Introducing electronic error-reporting dashboards or daily error briefings could create a culture of accountability and continuous improvement.

A strong correlation between the total sample volume and the error rate was found (p < 0.001) (Fig 2).

Approximately 70.8% of all errors occurred on high-volume days. This finding is consistent with practices observed in the Ethiopian and Saudi Arabian sites, where strain on operational hours became a major indicator for laboratory errors [8,9].

Similar patterns were observed during the COVID-19 pandemic, as a consequence of the overload of staff and the disruptions in the process, which aggravated the pre-analytical error rate [20].

The statistical significance of the workload–error relationship found in this study reinforces the idea that workload should be treated as a measurable risk factor in laboratory management rather than an unavoidable operational challenge.

The results also suggest that workforce fatigue, especially among technicians handling repetitive manual steps, can trigger cognitive errors, further aggravating pre-analytical inconsistency. Introducing regular micro-breaks, role rotation, and periodic skill assessment may reduce this burden.

Consequently, human factor engineering principles—such as ergonomic workspace design, scheduled rest periods, and supportive supervision—should be incorporated into laboratory policies to minimize error propagation.

Severity-based analysis yielded further insights into the laboratory workflow. Major errors predominantly occurred in the sample collection unit (48.2%), with errors mainly clustered around patient identification or sample integrity [7,21]. Moderate errors most frequently occurred in the biochemistry & immunology laboratories (77.1%); these processing errors were generally related to sample quality issues and procedural deviations**.**

This stratification of error severity allows laboratories to prioritize interventions based on clinical risk. For instance, major errors linked to incorrect labeling could have immediate consequences on treatment accuracy, while moderate errors may primarily affect turnaround times or require repeat sampling. Understanding this hierarchy helps allocate corrective resources efficiently.

**Fig 3:**
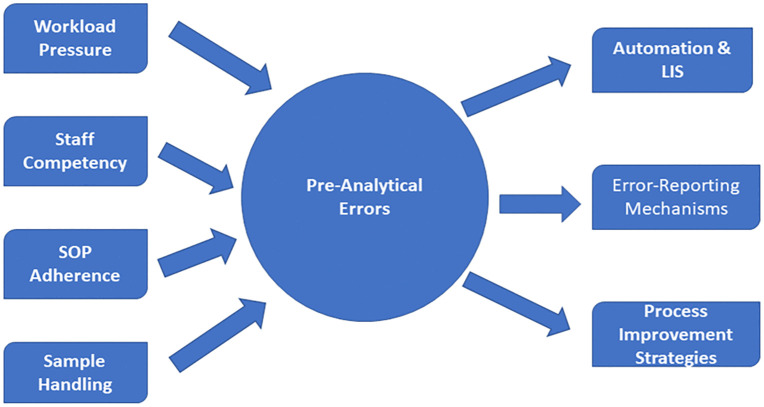
Conceptual framework of pre-analytical error contributors and proposed mitigation strategies.

The phlebotomy process and sample handling pose the highest risk of critical errors to frontline staff, underscoring how vulnerable this step is and the need for targeted interventions such as barcoding, automation, and deliberate staff education [22,23], as part of a comprehensive journey toward improved pre-analytical quality [24]. These key contributors and the corresponding mitigation strategies are summarized in a conceptual framework (Fig 3).

The components of this framework were informed by both the staff survey responses and established findings from previous literature.

Automation technologies such as pre-analytical workstations and digital barcode verification systems have already demonstrated measurable improvements in specimen traceability in other settings [22,23]. Introducing these systems gradually, even at a small scale, could yield substantial reductions in manual data entry errors. Moreover, competency-based training tailored to the pre-analytical phase should be institutionalized to build a long-term quality culture among laboratory technologists.

Implementing real-time quality indicators, continuous training sessions, and supervisory audits can help maintain compliance with ISO 15189:2022 recommendations. Process mapping and risk analysis tools such as Failure Mode and Effects Analysis (FMEA) may also provide structured pathways for reducing preventable incidents.

By integrating FMEA and root cause analysis into monthly quality meetings, laboratories can transition from a reactive to a preventive quality system. This proactive framework aligns with ISO 15189’s requirement for continual improvement and supports evidence-based decision-making within the laboratory management structure.

This study, while effective in many areas, has some limitations that must be acknowledged; one is that the study was conducted in a single centre, limiting the generalizability of the data collection. Also, since we did not have workload data by shift, the study may underrepresent the short-term workload effects in a clinical pathology laboratory. It is recommended for future studies to employ a multi-centre, real-time tracking of workload for validation of this data collection.

Future investigations could also explore the role of automation maturity, digital tracking infrastructure, and laboratory accreditation level as moderating factors influencing pre-analytical error frequency. Expanding this research to include rural diagnostic settings would provide a more holistic view of national laboratory quality challenges in Bangladesh.

## Conclusion

Pre-analytical errors, particularly in sample misplacement and incorrect labeling, continue to interfere with the quality of diagnostics in high-throughput laboratory settings [1,25]. This study confirms that high daily workloads and peak morning shifts are statistically significant drivers of error frequency, underscoring the operational strain on manual laboratory processes.

To improve process reliability, laboratory management should prioritize strategies such as automation, barcode tracking, structured training programs, and workload balancing, thereby reducing the risk to patient safety in resource-limited healthcare systems.

## Supporting information

S1 Table Staff survey responses showing the crosstabulation of shift timing and perceived workload. Morning-shift staff (94.1%, 16/17) more frequently reported a heavier workload compared to evening-shift staff (60.0%, 6/10).
(DOCX)

S2 Dataset Raw data underlying the findings described in the manuscript. (XLSX)
