# Supplementary material for: Pre-analytical errors in a high-volume Bangladeshi diagnostic centre: Prevalence, workload impact, and mitigation strategies
Source: PLoS One. 2026 Mar 4;21(3):e0341908. doi: 10.1371/journal.pone.0341908 (PMC12959673; doi:10.1371/journal.pone.0341908)
Supplement: S1 Table — Morning-shift staff (94.1%, 16/17) more frequently reported a heavier workload compared to evening-shift staff (60.0%, 6/10). (DOCX) [file pone.0341908.s001.docx]

**S1 Table: Staff survey responses on workload patterns**

| **Shift_Timing * Peak_Workload Crosstabulation** | | | | | |
| --- | --- | --- | --- | --- | --- |
|  | | | Peak_Workload | | Total |
|  |  |  | Yes | No |  |
| Shift_Timing | Morning | Count | 16 | 1 | 17 |
|  |  | % within Shift_Timing | 94.1% | 5.9% | 100.0% |
|  | Evening | Count | 6 | 4 | 10 |
|  |  | % within Shift_Timing | 60.0% | 40.0% | 100.0% |
| Total | | Count | 22 | 5 | 27 |
|  |  | % within Shift_Timing | 81.5% | 18.5% | 100.0% |
